# Metabolic Reprogramming Promotes Myogenesis During Aging

**DOI:** 10.3389/fphys.2019.00897

**Published:** 2019-07-10

**Authors:** Roberta Belli, Agnese Bonato, Luciana De Angelis, Simone Mirabilii, Maria Rosaria Ricciardi, Agostino Tafuri, Alessio Molfino, Stefania Gorini, Massimiliano Leigheb, Paola Costelli, Maurizia Caruso, Maurizio Muscaritoli, Elisabetta Ferraro

**Affiliations:** ^1^Department of Translational and Precision Medicine (Formerly Department of Clinical Medicine), Sapienza University of Rome, Rome, Italy; ^2^Institute of Cell Biology and Neurobiology, National Research Council (CNR), Rome, Italy; ^3^SAIMLAL, Histology Department, Sapienza University of Rome, Rome, Italy; ^4^Hematology, Sant’Andrea University Hospital, Department of Clinical and Molecular Medicine, Sapienza University of Rome, Rome, Italy; ^5^Laboratory of Cardiovascular Endocrinology, IRCCS San Raffaele Pisana, Rome, Italy; ^6^Department of Orthopaedics and Traumatology, Hospital “Maggiore della Carità”, Università del Piemonte Orientale (UPO), Novara, Italy; ^7^Department of Clinical and Biological Sciences, University of Turin, Turin, Italy

**Keywords:** metabolic reprogramming, myogenesis, trimetazidine, mitochondria, aging, neuromuscular activity, sarcopenia, metabolism

## Abstract

Sarcopenia is the age-related progressive loss of skeletal muscle mass and strength finally leading to poor physical performance. Impaired myogenesis contributes to the pathogenesis of sarcopenia, while mitochondrial dysfunctions are thought to play a primary role in skeletal muscle loss during aging. Here we studied the link between myogenesis and metabolism. In particular, we analyzed the effect of the metabolic modulator trimetazidine (TMZ) on myogenesis in aging. We show that reprogramming the metabolism by TMZ treatment for 12 consecutive days stimulates myogenic gene expression in skeletal muscle of 22-month-old mice. Our data also reveal that TMZ increases the levels of mitochondrial proteins and stimulates the oxidative metabolism in aged muscles, this finding being in line with our previous observations in cachectic mice. Moreover, we show that, besides TMZ also other types of metabolic modulators (i.e., 5-Aminoimidazole-4-Carboxamide Ribofuranoside-AICAR) can stimulate differentiation of skeletal muscle progenitors *in vitro*. Overall, our results reveal that reprogramming the metabolism stimulates myogenesis while triggering mitochondrial proteins synthesis *in vivo* during aging. Together with the previously reported ability of TMZ to increase muscle strength in aged mice, these new data suggest an interesting non-invasive therapeutic strategy which could contribute to improving muscle quality and neuromuscular communication in the elderly, and counteracting sarcopenia.

## Introduction

Loss of muscle mass and strength (often referred to as sarcopenia) are among the most relevant changes occurring in aging (Janssen et al., 2004; Lopez-Otin et al., 2013). Impairment of myogenesis contributes to sarcopenia; in fact, muscles of aged individuals display a low reservoir of resident skeletal muscle stem cells – satellite cells (SCs) – which are also characterized by impaired myogenic potential (Conboy et al., 2003; Carlson et al., 2009; Snijders et al., 2009; Shefer et al., 2010; Blau et al., 2015; Sousa-Victor and Munoz-Canoves, 2016). Moreover, mitochondrial dysfunctions play a key role in sarcopenia (Peterson et al., 2012; Hill and Van Remmen, 2014) and energy management is crucial also for SC fate; in particular, favoring the oxidative metabolism seems to enhance myogenic capabilities (Cerletti et al., 2012; Ryall, 2013; Ryall et al., 2015a). Although the precise mechanisms have to be elucidated, during the transition from quiescence to activation, proliferation, and differentiation, SCs have to meet specific bioenergetic demands and undergo metabolic changes which regulate chromatin accessibility and transcription (Cervelli et al., 2009; Ryall, 2013; Ryall et al., 2015b; Ryall and Lynch, 2018). Moreover, slow-oxidative myofibers are associated to more SCs and capillaries compared to fast-glycolytic myofibers (Christov et al., 2007; Yin et al., 2013), and high local oxygen concentration stimulates SC differentiation (Christov et al., 2007).

Based on these premises, we hypothesize that an optimization of energy production through metabolic reprogramming might be an interesting strategy for both counteracting mitochondrial dysfunctions and supporting SC function, so improving myogenesis in aging. In support of this hypothesis, we have previously shown that the metabolic modulator trimetazidine (TMZ) – shifting the energy metabolism from fatty acid to glucose oxidation, thus enhancing metabolic efficiency (Jaswal et al., 2011; Zhao et al., 2016) – increases muscle strength in aged mice and stimulates myoblast differentiation *in vitro* (Ferraro et al., 2016; Gatta et al., 2017). The main aim of this study was to investigate the effect of TMZ on myogenesis in aging. Moreover, other metabolic modulators were tested for their ability to improve the myogenic potential of SC *in vitro*.

## Materials and Methods

### Animals

Mice were cared for in compliance with the Italian Ministry of Health Guidelines (n° 86609 EEC, permit number 106/2007-B) and the Policy on Human Care and Use of Laboratory Animals (NIH 1996) (Ferraro et al., 2016). 22-month-old C57BL6/J male mice were divided into two groups (*n* = 6 each); one group received 5 mg/kg TMZ (Alfa Aesar Thermofisher) by intraperitoneal injection twice a day for 12 days, the control group received PBS injections. Balb-c mice were inoculated dorsally with C26 colon-carcinoma cells and treated or not (*n* = 7 per group) with 5 mg/kg TMZ once a day for 12 days (Molinari et al., 2017). Healthy Balb-c mice administered or not TMZ (*n* = 6 per group) were used as controls. 12 days after TMZ-treatment the animals were sacrificed and gastrocnemius, tibialis anterior and hearts were excised and stored at −80°C.

### Western Blotting

Gastrocnemius and hearts lysates (obtained by centrifugation of homogenates) were separated by SDS-PAGE, transferred to nitrocellulose membranes and probed using antibodies against MyoD, Myogenin (M3512, M3559-Dako), Desmin, Collagen type-III, α-SMA (D1033, C7805, A5228-Sigma-Aldrich), PGC1α, β-ATPase (AB3242, MAB3494-Millipore), TFAM, Tom20, CSQ2, fsTn-I, VEGF, VE-Cadherin, PECAM, SDHA and PPARγ (sc-23588, sc-11415, sc-390999, sc-377382, sc-7269, sc-9989, sc-46694, sc-377302, sc-7273-Santa Cruz Biotechnology), CoxIV (Ab14744-Abcam) and the appropriate secondary antibodies. α-tubulin or actin (T5168, A3853-Sigma-Aldrich) were used for normalization. Quantification was performed as previously described (Ferraro et al., 2016).

### Quantitative RT-PCR

RNA isolation was performed as previously described (Ferraro et al., 2016). cDNA was synthesized with the GoScript Reverse Transcription System (Promega). qRT-PCR was performed with the SYBR-green master mix (Promega) by using an Applied Biosystem Step One PCR system. Data were normalized to 18S and analyzed using the comparative CT (2-ΔΔCT) method.

### C2C12 Cell Culture, Satellite Cells Isolation, and Treatments

C2C12 skeletal myoblasts were cultured in growth medium (GM; DMEM supplemented with 20% FBS). Before confluency the GM was replaced with differentiation medium (DM; supplemented with 2% horse serum) with or without AICAR (Enzo Life Sciences). As for SCs isolation, the manufacturer (Miltenyi Biotec)’s instructions were followed (Gatta et al., 2017). The purified cells were then resuspended in DMEM supplemented with 10% FBS, 20% HS, and 3% CEE, and seeded in collagen precoated dishes.

### Fluorescence Microscopy

Cells or Tibalis anterior cross-sections were immunostained as previously described (De Luca et al., 2013) with anti-MyHC (MF20-DSHB), Myogenin (sc-576), Pax7 (DSHB), and PECAM antibodies overnight at 4°C. Fluorofore-conjugated secondary antibodies were applied 1 h at RT and nuclei were counterstained with Hoechst.

### Metabolic Analysis

Metabolic analysis was performed using the Seahorse-XF24 Flux Analyzer (Agilent Technologies). C2C12 cells were seeded onto XF24 plates. The following day 10 μM TMZ was added. On the day of the analysis, the medium was replaced with XF-Base medium ± 10 μM TMZ and plates were incubated for 30 min at 37°C in a CO_2_-free incubator. Oxygen Consumption Rate (OCR) was measured for the basal state, and followed by the sequential injection of oligomycin, FCCP and a AntimycinA+Rotenone (Berghella and Ferraro, 2012; Divakaruni et al., 2014).

### Sirius Red Staining

Tibialis anterior cryo-sections fixed with Bouin’s solution (Sigma-Aldrich) for 1 h were stained with Picro-Sirius Red dye (Sigma-Aldrich) for 1 h.

### Statistical Analysis

Data are presented as mean ± standard error (SEM). Distribution of data was checked by Kolmogorov–Smirnov test and Shapiro–Wilk normality test. For normally distributed data, statistical differences between two groups were verified by Student’s *t*-test (two-tailed). For non-parametric variables, groups were compared by Mann–Whitney *U* test. For the comparison between more than two groups Kruskal–Wallis test was performed. *P* < 0.05 was considered significant. The statistical analyses were performed using SPSS 25.0 (SPSS Inc., 2019).

## Results

### Metabolic Reprogramming Stimulates Myogenic and Oxidative Gene Expression in the Skeletal Muscle of Aged Mice

**FIGURE 1:**
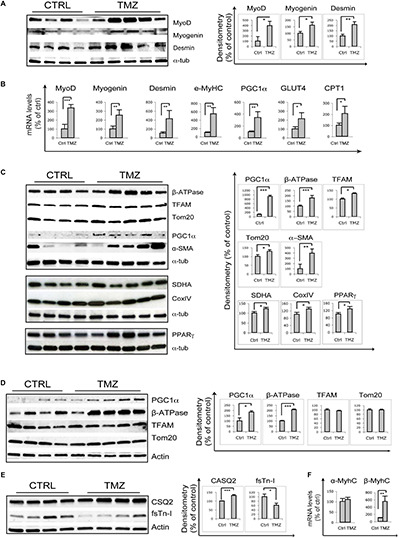
Trimetazidine stimulates myogenic and mitochondrial gene expression. **(A)** Gastrocnemius extracts from untreated aged mice (Ctrl) and TMZ-treated aged mice (TMZ) were assayed for MyoD Myogenin and Desmin protein levels, **(B)** for the mRNA levels of MyoD, Myogenin, Desmin, MyHC, PGC1α, GLUT4, and CPT1 which were evaluated by qRT-PCR, and **(C)** for β-ATPase, TFAM, Tom20, PGC1α, and α-SMA protein levels. Protein levels of representative four out of six untreated mice and five out of six TMZ-treated mice are shown. **(D)** Heart extracts from untreated aged mice (Ctrl) and TMZ-treated aged mice (TMZ) were assayed for PGC1α, β-ATPase, TFAM, and Tom20 protein levels and **(E)** for CSQ2 and fsTn-I protein levels. Protein levels of representative four out of six untreated mice and 4/5 out of six TMZ-treated mice are shown. **(F)** Heart RNA extracted from untreated aged mice (Ctrl) and TMZ-treated aged mice (TMZ) was assayed for the mRNA levels of α-MyHC and β/slow-MyHC which were evaluated by qRT-PCR. In all WB density of immunoreactive bands was calculated using the ImageQuant TL software from GE Healthcare Life normalized for α-tubulin or actin used as loading control. Each value indicates the mean ± SEM (reported as percentage of Ctrl) of the densitometric analysis on three independent immunoblots. In all qRT-PCRs, data were normalized to 18S ribosomal RNA used as internal control. Data display the percentage of mRNAs relative to control. Data shown are the mean ± SEM from three experiments each performed in duplicate. For all experiments ^*^p < 0.05, *^∗∗^p* < 0.01, and ^∗∗∗^*p* < 0.001 by Student′s *t*-test or Mann–Whitney *U* test as appropriate. Primers used: 18S: Fw-5′-CCCTGCCCTTTGTACACACC-3′ Rv-5′-CGA TCCGAGGGCCTCACTA-3′; MyoD Fw-5′-CCCCGGCGGCAGAATGGCTACG-3′ Rv-5′-GGTCTGGGTTCCCTGTTCTGTG-3′; Myogenin: Fw-5′-GGGCCCCTGGAA GAAAAG-3′ Rv-5′-AGGAGGCGCTGTGGGAGT-3′; Desmin: Fw-5′-GAGGTTGTCAGCGAGGCTAC-3′ Rv-5′-GAAAAGTGGCTGGGTGTGAT-3′; MyHC Fw-5′-CAAG TCATCGGTGTTTGTGG-3′ Rv-5′-TGTCGTACTTGGGAGGGTTC-3′; CTP1: Fw-5′-CCCATGTGCTCCTACCAGAT-3 Rv-5-CCTTGAAGAAGCGACCTTTG-3; PGC1α: Fw-5′-GTCAACAGCAAAAGCCACAA-3 Rv-5′-TCTGGGGTCAGAGGAAGAGA-3′; GLUT-4: Fw-5′-GGCATGGGTTTCCAGTATGT-3′ Rv-5′-GCCCCTCAGTCATTC TCATG-3′; α-MyHC: Fw-5′-AACAACCCATACGACTACGCC-3′ Rv-5′-CAGCATCTTCTGTGCCATCA-3′; β/slow-MyHC Fw-5′-TGCAGCAGTTCTTCAACCAC-3′ Rv-5′-TCGAGGCTTCTGGAAGTTGT-3′; VEGF: Fw-5′-CTGTGCAGGCTGCTGTAACG-3′ Rv-5′-GTTCCCGAAACCCTGAGGAG-3′; MyHC: Fw-5′-TCGTCTCGCTT TGGCAA-3′ Rv-5′-TGGTCGTAATCAGCAGCA-3′.

Skeletal muscle of 22-month-old mice treated with the metabolic modulator TMZ for 12 days were assayed for the expression of myogenic markers by qRT-PCR and WB analysis which revealed MyoD, Myogenin, Desmin, and eMHC up-regulation following TMZ treatment (Figures 1A,B). Moreover, TMZ stimulated the expression of mitochondrial and oxidative metabolism markers such as mitochondrial transcription factor A (TFAM) and mitochondrial protein Tom20 (Figure 1C). This is in line with the observed TMZ-mediated induction of peroxisome proliferator-activated receptor gamma (PPARγ)-coactivator-1-alpha (PGC1α), a master regulator of mitochondrial biogenesis also involved in myogenesis (Figures 1B,C; Haralampieva et al., 2017, 2018). Moreover, the up-regulation of mitochondrial ATP-synthase β-subunit (β-ATPase), succinate-dehydrogenase complex subunit-A (SDHA), cytochrome-*c*-oxidase (CoxIV), and PPARγ (Figure 1C) were indicative of a reprogramming toward a more oxidative metabolism. Increase of Glut4 transcript levels is also triggered by TMZ (Figure 1B), consistently with our previous data showing that this drug enhances glucose uptake (Ferraro et al., 2013, 2016).

Overall, these data confirm that, in sarcopenic skeletal muscles, TMZ is able to modulate the levels of markers of myogenesis and mitochondrial homeostasis.

Interestingly, we also observed a marked TMZ-dependent increase of α-smooth muscle actin (α-SMA) in aged muscles (Figure 1C), as well as in muscles of tumor-bearing cachectic mice (Supplementary Figure S1). α-SMA over-expression could reflect enhanced capillarization, which would be consistent with TMZ-induced up-regulation of vascular endothelial growth factor (VEGF), as reported in Supplementary Figure S2A. VEGF protein levels also tended to increase, although not significantly (Supplementary Figure S2B). However, VEGF induction was not associated with increased capillarization, as evaluated by PECAM staining and by vascular endothelial (VE)-cadherin WB detection (Supplementary Figure S2C and data not shown).

α-SMA is also a marker of myofibroblasts which contribute to fibrosis (Li and Huard, 2002); however, the positivity to Sirius Red staining was not different between TMZ-treated and untreated muscles (Supplementary Figure S2D). This result, together with the observation that Collagen III levels were not influenced by TMZ (Supplementary Figure S2A) rule out an effect of the drug on fibrosis

Since TMZ has been extensively studied for its effect on myocardium (Jaswal et al., 2011; Zhao et al., 2016), we also evaluated TMZ’s effect on cardiac mitochondrial proteins, and we found increased expression of PGC1α and β-ATPase, whereas no significant changes were observed on TFAM and Tom20 (Figure 1D). The stronger TMZ’s effect on skeletal muscle mitochondria compared to cardiac ones might be explained by the higher plasticity of the former tissue compared to the latter, resulting in a fast response to environmental stimuli. Interestingly, we also found that, in the heart, TMZ stimulated the expression of the sarcoplasmic reticulum Ca^2+^-binding protein calsequestrin-2 (CSQ2) and of the slow-twitch myosin heavy chain β-isoform (β/slow-MyHC) while reducing the expression of the fast isoform of troponin-I (fsTn-I) and having no effect on the fast α-MyHC (Figures 1E,F). Since we have recently shown that TMZ stimulates a fast-to-slow myofiber switch (Molinari et al., 2017), it is worth mentioning that the CSQ2 isoform, similarly to β/slow-MyHC are typically expressed in cardiomyocytes as well as in slow-twitch skeletal muscles.

### Similarly to TMZ, Other Metabolic Modulators Can Potentiate Myogenesis

**FIGURE 2:**
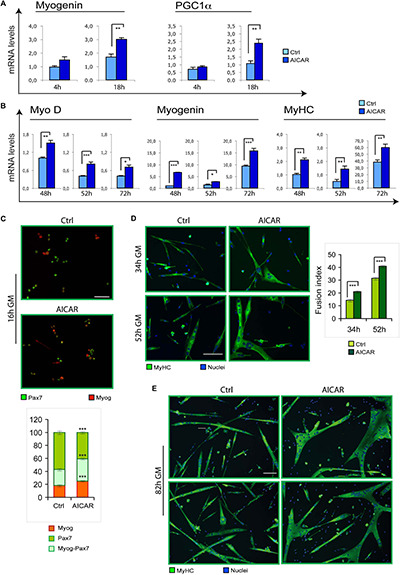
Similarly to TMZ, also AICAR stimulates myogenic differentiation. **(A)** The mRNA levels of Myogenin and PGC1a were evaluated by quantitative real time PCR in C2C12 myoblasts differentiating for 4 and 18 h with or without 0.5 mM AICAR. The mRNA expression values were normalized to those of 18S ribosomal RNA, used as internal control, and are displayed as folds relative to a plate reference control sample (0 h in differentiation medium, not shown). Data shown are the mean ± SEM from four experiments each performed in triplicate. ^∗∗^*p* ≤ 0.01 by two-tailed Student’s *t*-test. **(B)** The mRNA levels of MyoD, Myogenin, and MyHC were evaluated by quantitative real time PCR in primary satellite cells cultured for 24 h and then treated with 0.5 mM AICAR for further 24 h (total 48 h in culture), or cultured for 48 h and then treated with 0.5 mM AICAR for further 4 or 24 h (total 52 or 72 h in culture, respectively). The mRNA expression values were normalized to 18S RNA used as internal control, and are displayed as folds relative to a plate reference control sample (48 h in culture without AICAR treatment). Data shown are the mean ± SEM from three experiments, each performed in triplicate. ^*^p < 0.05, ^∗∗^*p* ≤ 0.01, and ^∗∗∗^*p* ≤ 0.005 by two-tailed Student’s *t*-test. **(C)** Satellite cells were cultured for 6 h and then treated with or without 0.5 mM AICAR for 10 h before fixation and co-immunostaining for myogenin (red) and Pax7 (green). The graph represents the quantification of myogenin^+^/Pax7^–^ , myogenin^+^/Pax7^+^, or myogenin^–^ /Pax7^+^ cells. Data are the means ± SEM from three experiments and are expressed as the percentage in each category of the total positive cells. For each experiment, at least 10 microscopy fields were analyzed for a total of at least 1000 cells counted for each condition. **(D,E)** Satellite cells were cultured in 35 mm collagen-coated dishes for 12 h and then treated with or without 0.5 mM AICAR for 22, 40, or 70 h before fixing and immunostaining for MyHC (green). Counterstaining with Hoechst was used to visualize all nuclei (blue). The graph represents the quantification of fusion index at 34 and 52 h. Data are from three independent experiments. Values are expressed as the mean ratio ± SEM of nuclei present in myotubes, containing at least three nuclei, to the total number of nuclei. Asterisks denote significance (^∗∗^*p* < 0.01, ^∗∗∗^*p* < 0.001). Scale bar: 50 μm.

To evaluate if myogenesis can be stimulated by other metabolic modulators, we tested the effect of an activation of AMPK by 5-Aminoimidazole-4-Carboxamide Ribofuranoside (AICAR). Indeed, metabolic reprogramming driven by AICAR could influence skeletal myoblast differentiation *in vitro*. Specifically, while high concentrations of AICAR (2 mM) resulted toxic for C2C12 myoblasts (Supplementary Figure S3), 0.5 mM AICAR induced the differentiation marker myogenin and PGC1α (Figure 2A). Corroborating these findings, we found that differentiation of SC cultures was enhanced by AICAR, as demonstrated by increased MyoD, myogenin and MyHC mRNA levels (Figure 2B). Furthermore, co-immunostaining for myogenin and the SC marker Pax7 revealed a precocious onset of differentiation triggered by AICAR, as indicated by an increase in the fraction of cells expressing myogenin or co-expressing myogenin and Pax7, with a reciprocal decrease of only Pax7-positive cells (Figure 2C). At later stages of myogenesis, immunostaining for MyHC revealed, for AICAR-treated cells, a fusion index higher compared to controls and much larger myotubes (Figures 2D,E).

Based on the above data and on our previous results showing stimulation of *in vitro* myoblast differentiation by TMZ (Gatta et al., 2017), and taking into account that myogenic differentiation is associated with an increased mitochondrial mass (Moyes et al., 1997; Barbieri et al., 2011), we assessed the rate of respiration of C2C12 myoblasts undergoing differentiation with and without TMZ. Unexpectedly, the OCR during myoblast differentiation was similar in both conditions (Supplementary Figures S4A,B). A trend toward increased oxygen consumption was only observed when myoblasts were cultured in GM without pyruvate and were acutely treated with TMZ for 20 min (Supplementary Figures S4C,D).

## Discussion

The present study demonstrates the ability of TMZ to potentiate myogenesis in aged muscle. This correlates and potentially explains the high number of centro-nucleated myofibers and a shift toward smaller cross-sectional areas we have previously observed after TMZ treatment in aged mice, (Ferraro et al., 2016). Moreover, our findings are also in line with the ability of TMZ to enhance myoblast differentiation *in vitro* and in cachectic tumor-bearing mice (Gatta et al., 2017).

Our results also reveal that TMZ up-regulates mitochondrial proteins and stimulates the oxidative metabolism in aged mice, similarly to previous observations in cachectic mice (Moyes et al., 1997; Barbieri et al., 2011; Molinari et al., 2017). Carnitine palmitoyltransferase-I (CPT1) up-regulation suggests a metabolic compensative response to TMZ-induced fatty acid β-oxidation reduction (Figure 1B).

Particularly interesting, although subject to various interpretations, is the marked α-SMA induction triggered by TMZ (Figure 1C and Supplementary Figure S1). α-SMA is a marker of perycites, cells with supportive function strictly associated to the microvessel endothelium and abundant in angiogenic sprouts (Armulik et al., 2005). The increase of α-SMA expression could suggest enhanced capillarization triggered by TMZ, which would be consistent with VEGF up-regulation, although VE-cadherin and PECAM levels do not change upon TMZ treatment. We hypothesize that 12 days of TMZ treatment might be enough to induce VEGF but not its downstream effects on capillarization. Moreover, pericytes significantly contribute to skeletal myogenesis and α-SMA is also expressed in myoblasts (Dellavalle et al., 2007, 2011; Cappellari and Cossu, 2013), this being in line with the observed stimulation of myogenesis by TMZ. Additionally, during adult skeletal muscle regeneration, some regenerating fibers express α-SMA (Chaponnier and Gabbiani, 2016).

Here we also show that, besides TMZ, also the metabolic reprogramming agent AICAR can promote myoblast differentiation. Similarly, ranolozine has been proposed as having the same effect (Terruzzi et al., 2017). Notably, in contrast to our data, AICAR has previously been shown to inhibit C2C12 differentiation (Williamson et al., 2009) These contradictory results might be due to the different AICAR concentrations used, indeed, here we demonstrated that high AICAR concentrations are toxic for myoblasts.

Finally, we report a trend to oxygen consumption increase in TMZ-treated myoblasts cultured in GM and without pyruvate, possibly because the presence of high pyruvate concentration in the medium might mask the metabolic shift from fatty acid to glucose oxidation triggered by TMZ which increases pyruvate. Similarly, the DM would hide a mild stimulatory effect on differentiation.

Overall, these data confirm the occurrence of a clear effect of TMZ, and potentially of other modulators of the metabolism, both on myogenesis and on mitochondria homeostasis. Given that TMZ also increases muscle strength in aged animals (Ferraro et al., 2016), this and other metabolic modulators appear promising as therapeutic agents, thanks to their potential to counteract sarcopenia and improve neuromuscular activity, thus increasing elderly people’s self-sufficiency.

## Author Contributions

RB and EF designed the experiments. RB, AB, LD, MR, SM, and SG performed the experiments and analyzed data. RB, AB, LD, MR, SM, and EF interpreted the data. AT, MC, and AM contributed to the development of the study by the provision of study material and data interpretation. AM, MM, PC, ML, and EF wrote the manuscript and provided the financial support. EF conceived the study. All authors contributed to the manuscript revision, read, and approved the final version of the manuscript for submission.

## Conflict of Interest Statement

The authors declare that the research was conducted in the absence of any commercial or financial relationships that could be construed as a potential conflict of interest. The handling Editor declared a past co-authorship with one of the authors, EF.
