# Corrigendum: Metabolic Reprogramming Promotes Myogenesis During Aging

**DOI:** 10.3389/fphys.2021.674698

**Published:** 2021-05-12

**Authors:** Roberta Belli, Agnese Bonato, Luciana De Angelis, Simone Mirabilii, Maria Rosaria Ricciardi, Agostino Tafuri, Alessio Molfino, Stefania Gorini, Massimiliano Leigheb, Paola Costelli, Maurizia Caruso, Maurizio Muscaritoli, Elisabetta Ferraro

**Affiliations:** ^1^Department of Translational and Precision Medicine (Formerly Department of Clinical Medicine), Sapienza University of Rome, Rome, Italy; ^2^Institute of Cell Biology and Neurobiology, National Research Council (CNR), Rome, Italy; ^3^SAIMLAL, Histology Department, Sapienza University of Rome, Rome, Italy; ^4^Hematology, Sant'Andrea University Hospital, Department of Clinical and Molecular Medicine, Sapienza University of Rome, Rome, Italy; ^5^Laboratory of Cardiovascular Endocrinology, IRCCS San Raffaele Pisana, Rome, Italy; ^6^Department of Orthopaedics and Traumatology, Hospital “Maggiore della Carità”, Università del Piemonte Orientale (UPO), Novara, Italy; ^7^Department of Clinical and Biological Sciences, University of Turin, Turin, Italy

**Keywords:** metabolic reprogramming, myogenesis, trimetazidine, mitochondria, aging, neuromuscular activity, sarcopenia, metabolism

In the original article, we neglected to include the funder Ricerca Finalizzata Grant (RF-2010-231-8508) to Elisabetta Ferraro.

In the published article, Stefania Gorini was not included as an author and she should have the affiliation Laboratory of Cardiovascular Endocrinology, IRCCS San Raffaele Pisana, Rome, Italy.

Stefania Gorini was not included as an author in the published article. The corrected Author Contributions Statement appears below.

RB and EF designed the experiments. RB, AB, LD, MR, SM, and SG performed the experiments and analyzed data. RB, AB, LD, MR, SM, and EF interpreted the data. AT, MC, and AM contributed to the development of the study by the provision of study material and data interpretation. AM, MM, PC, ML, and EF wrote the manuscript and provided the financial support. EF conceived the study. All authors contributed to the manuscript revision, read, and approved the final version of the manuscript for submission.

The authors apologize for this error and state that this does not change the scientific conclusions of the article in any way. The original article has been updated.

