# Supplementary material for: Metabolic Reprogramming Promotes Myogenesis During Aging
Source: Front Physiol. 2019 Jul 10;10:897. doi: 10.3389/fphys.2019.00897 (PMC6636331; doi:10.3389/fphys.2019.00897)
Supplement: Supplementary file 1 [file Data_Sheet_1.pdf]

Figure S1

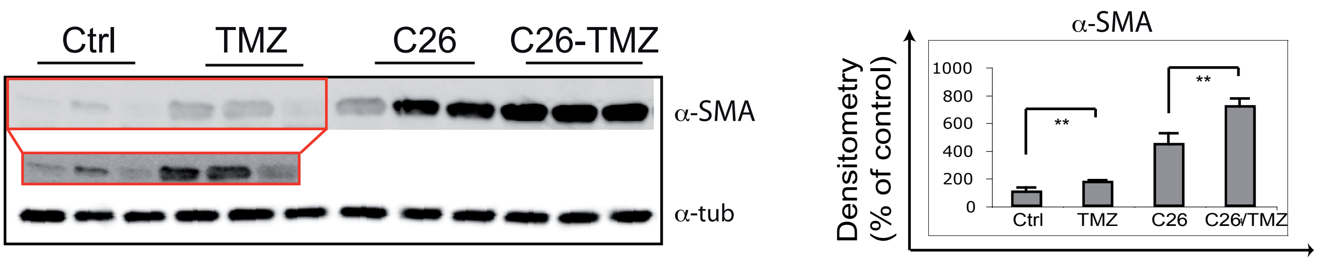

Figure S1 TMZ-dependent up-regulates α-SMA in skeletal muscle of C26 tumor-bearing cachectic mice. Gastrocnemius extracts from untreated control mice (Ctrl), TMZ-treated control mice (TMZ), C26 tumor-bearing mice (C26) and TMZ-treated C26 mice (C26-TMZ) were assayed for α-SMA protein levels. Protein levels of representative 3-4 out of 6-7 mice are shown. $^{**}p < 0.01$ by Kruskal-Wallis test .

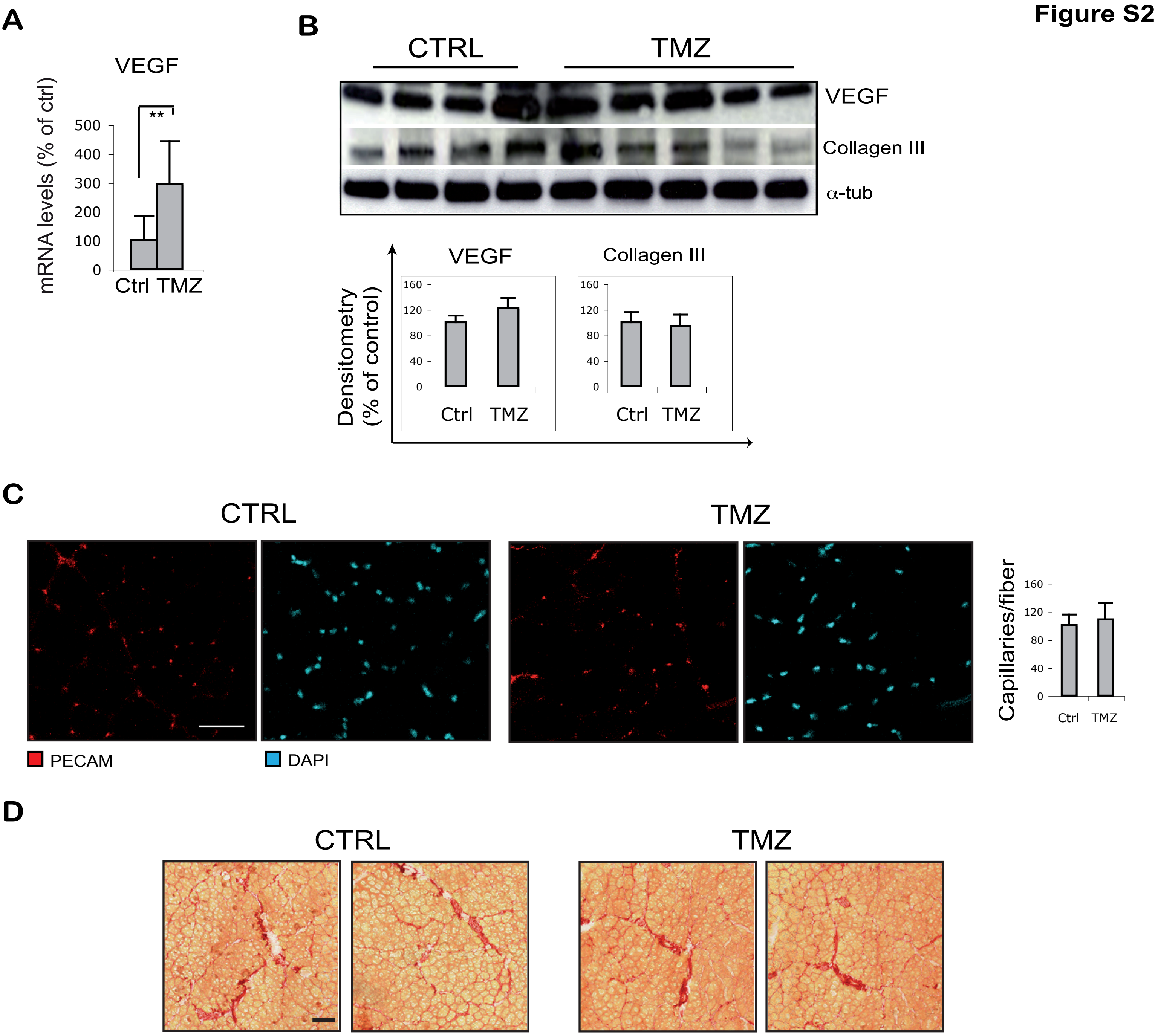

Figure S2 Effect of TMZ on capillarization and fibrosis in aging.

(A) Gastrocnemius extracts from untreated aged mice (Ctrl) and TMZ-treated aged mice (TMZ) were assayed for the mRNA levels of VEGF evaluated by qRT-PCR. Data were normalized to18S ribosomal RNA used as internal control. Data shown are the mean ± s.e.m.from two experiments each performed in triplicate. (B) GSN extracts from untreated aged mice (Ctrl) and TMZ-treated aged mice (TMZ) were assayed for VEGF and Collagen type-III protein levels. Protein levels of representative 4 out of 6 untreated mice and 5 out of 6 TMZ-treated mice are shown. α-tubulin was used as loading control. (C) Representative images of immunofluorescence staining on tibialis anterior (TA) cross-cryosections for the detection of PECAM (red) and nuclei (DAPI) in aged mice (Ctrl) and TMZ-treated aged mice (TMZ). Calculations were performed on three untreated mice and three TMZ-treated mice and at least 5 section from each mouse were evaluated. Percentage of the PECAM stained capillaries normalized to the number of myofibers in each cryosection are shown in the histogram where the Ctrl is arbitrarily set to 100 and means±s.e.m are reported. Scale bar: 50 μm. (D) Sirius red staining (specific for collagen isoforms) of TA cryosections.Three untreated mice and three TMZ-treated mice and at least 5 section from each mouse were evaluated. Scale bar: 50 μm.

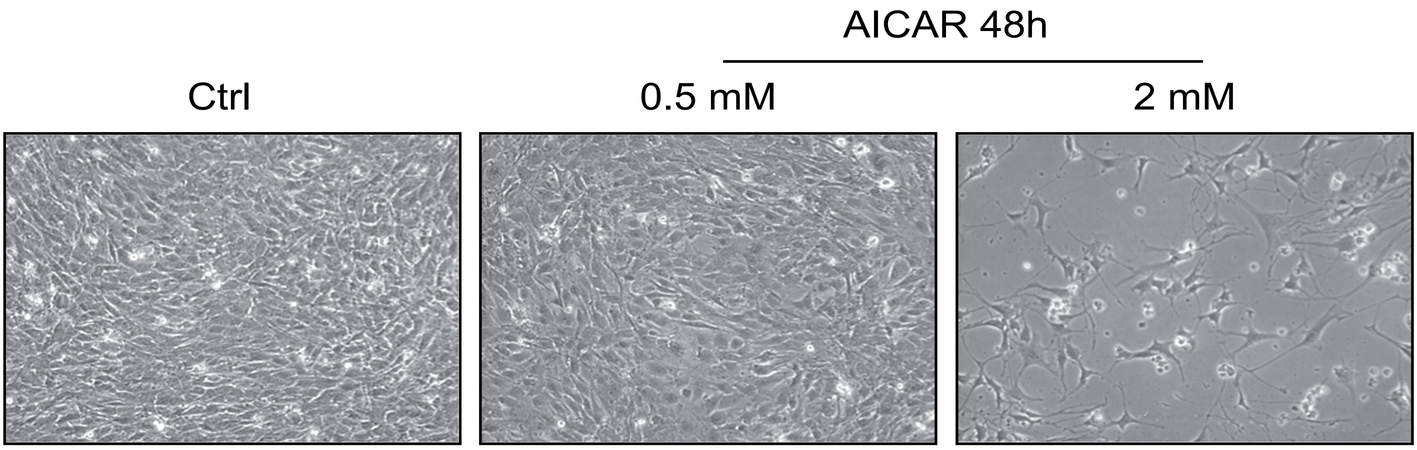

Figure S3 High concentrations of AICAR (2 mM) resulted toxic for C2C12 myoblasts.
Brightfield image of C2C12 myoblasts untreated (Ctrl) and treated for 48 h with 0.5 mM or 2 mM AICAR.

Figure S4

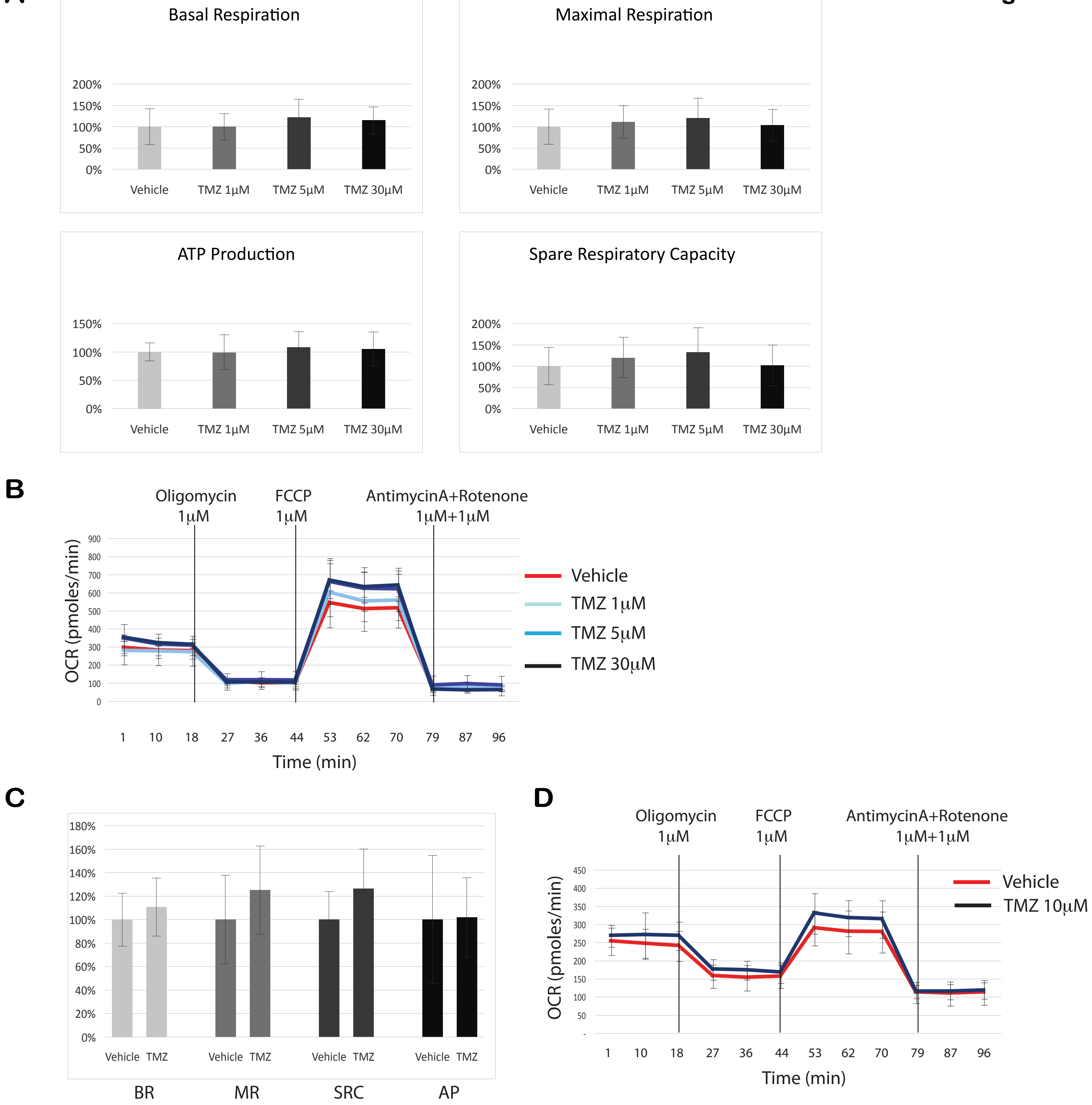

Figure S4 Mitochondrial respiration of differentiating C1C12 treated with TMZ.
(A) Mitochondrial respiration parameters deriving from mito stress test experiments and **(B)** – representative time course of a mito stress test on differentiating C1C12 exposed to the indicated TMZ concentrations. C2C12 cells were seeded onto XF24 plates (9000 cells/well) in DMEM-GlutaMAX, serum and pyruvate (GM). The following day the medium was replaced with DM ±10uM TMZ for 2h/24h (each condition in quadruplicate). After the incubation, on the day of the analysis, the medium was replaced with XF-Base medium (unbuffered DMEM medium supplemented with 2mM L-glutamine, 11mM Glucose and 1.2mM Pyruvate, pH 7.35) ±10uM TMZ and plates were incubated for 30 min at 37°C in a CO2-free incubator. **(C)** Mitochondrial respiration parameters deriving from mito stress test experiments and **(D)** representative time course of a mito stress test on differentiating C1C12 exposed to 10uM TMZ concentrations. C2C12 cells were seeded onto XF24 plates (9000 cells/well) in DMEM-GlutaMAX and serum without pyruvate. The following day the medium was replaced with the same medium ±10uM TMZ for 20 min (each condition in quadruplicate). After the incubation, on the day of the analysis, the medium was replaced with XF-Base medium (unbuffered DMEM medium supplemented with 2mM L-glutamine, 11mM Glucose pH 7.35) with1.2mM Pyruvate (A, B) or without pyruvate (C,D), ±10uM TMZ and plates were incubated for 30 min at 37°C in a CO2-free incubator. BR = Basal Respiration; MR = Maximal Respiration; AP = ATP Production; SRC = Spare Respiratory Capacity. Parameters are calculated as per manufacturer's instruction (Divakaruni AS et al. 2014. PMID: 25416364).
